# How to Obtain a Reliable Estimate of Occupational Exposure? Review and Discussion of Models’ Reliability

**DOI:** 10.3390/ijerph16152764

**Published:** 2019-08-02

**Authors:** Andrea Spinazzè, Francesca Borghi, Davide Campagnolo, Sabrina Rovelli, Marta Keller, Giacomo Fanti, Andrea Cattaneo, Domenico Maria Cavallo

**Affiliations:** Dipartimento di Scienza e Alta Tecnologia, Università degli Studi dell’Insubria, Via Valleggio 11, 22100 Como, Italy

**Keywords:** occupational exposure assessment, Advanced REACH Tool (ART), ECETOC TRA, STOFFENMANAGER^®^, TREXMO, MEASE, EMKG-Expo-Tool, accuracy, REACH

## Abstract

Evaluation and validation studies of quantitative exposure models for occupational exposure assessment are still scarce and generally only consider a limited number of exposure scenarios. The aim of this review was to report the current state of knowledge of models’ reliability in terms of precision, accuracy, and robustness. A systematic review was performed through searches of major scientific databases (Web of Science, Scopus, and PubMed), concerning reliability of Tier1 (“ECETOC TRA”-European Centre for Ecotoxicology and Toxicology of Chemicals Targeted Risk Assessment, MEASE, and EMKG-Expo-Tool) and Tier2 models (STOFFENMANAGER^®^ and “ART”-Advanced Registration, Evaluation, Authorization and Restriction of Chemicals (REACH) Tool). Forty-five studies were identified, and we report the complete information concerning model performance in different exposure scenarios, as well as between-user reliability. Different studies describe the ECETOC TRA model as insufficient conservative to be a Tier1 model, in different exposure scenarios. Contrariwise, MEASE and EMKG-Expo-Tool seem to be conservative enough, even if these models have not been deeply evaluated. STOFFENMANAGER^®^ resulted the most balanced and robust model. Finally, ART was generally found to be the most accurate and precise model, with a medium level of conservatism. Overall, the results showed that no complete evaluation of the models has been conducted, suggesting the need for correct and harmonized validation of these tools.

## 1. Introduction

To comply with the REACH (Registration, Evaluation, Authorization and Restriction of Chemicals) regulation (EC 1907/2006), manufacturers and importers of chemical substances must conduct quantitative occupational exposure studies for identified and selected exposure scenarios. The exposure assessment process can be based (1) on measured exposure data or (2) on the use of exposure assessment tools. The use of exposure modeling for the assessment of exposure to chemicals by inhalation is also considered in a recent EU standard (EN 689: 2018) that request of appraisers to produce reliable exposure estimates using appropriate and validated models or algorithms.

When exposure modeling is used, the European Chemical Agency (ECHA) suggests the use of exposure-specific tools for the evaluation of exposure assessment, following a tiered approach. The tiered approach involves the use of simplified and conservative exposure models in the first step of the evaluation (Tier1), followed, if necessary, by using more complex and detailed (Tier2) models [1]. Available exposure models vary by domain of applicability, level of detail, and type of results output [2]. Despite these differences, an integrated methodology has yet to be defined (e.g., different tools require different scenario characterization, data input, data management, and different results formats). Evaluation and validation studies of Tier1 and Tier2 exposure models, as well as studies on their reliability (in terms of precision, accuracy, and robustness), are still scarce and generally only consider a limited number of exposure scenarios [3,4]. The validation and evaluation of models in different exposure conditions and scenarios are particularly relevant, as exposure scenarios can be affected by many uncertainties, introducing some randomness in the input parameters [3,5], which reduces the precision of the model [6]. Concerning model precision, the between-user reliability issue in exposure estimates that it is a problem that cannot be neglected [7,8,9].

Thus, to define the current available evidence about the reliability of inhalation occupational exposure estimation models, a systematic review was performed, with the aims to: (1) identify whether the scientific literature regarding validation and evaluation of occupational exposure models is exhaustive, (2) identify which kind of evaluation studies have not yet been conducted, (3) identify which scenarios have not yet been considered in model evaluation studies, and (4) provide useful information to users regarding the choice of the best model to use in a particular scenario. Exposure assessment tools considered in this review are those suggested by the European Chemicals Agency (ECHA) [1] as Tier 1, including European Centre for Ecotoxicology and Toxicology of Chemicals Targeted Risk Assessment (ECETOC TRA), MEASE, and the EMKG Expo-Tool, or Tier2, including STOFFENMANAGER^®^ (www.stoffenmanager.com; Cosanta BV, Amstelveen, The Netherlands) and the Advanced REACH Tool (ART; www.advancedreachtool.com). The considered models are briefly described in Section 3.1.1. (Tier1 models) and Section 3.1.2. (Tier2 models). Although it is not a stand-alone model, the Translation Exposure Model (TREXMO) was also included in the review (Section 3.1.3.), as several studies concerning this tool were collected during our systematic research, the contents of which were considered relevant for dealing with the issue of model reliability.

## 2. Materials and Methods

This study involved a systematic review process conducted according to the Preferred Reporting Items for Systematic Reviews and Meta-Analyses Statement criteria (PRISMA) [10]. Three of the principal scientific databases (Scopus, PubMed, and ISI Web of Knowledge) were searched for the identification and selection of studies that addressed the reliability of occupational exposure modeling tools. Keywords and the query structure were arranged as a function of the writing rules required by the selected databases, but the list of keywords used was the same for the three databases (Table 1).

**Figure 1:**
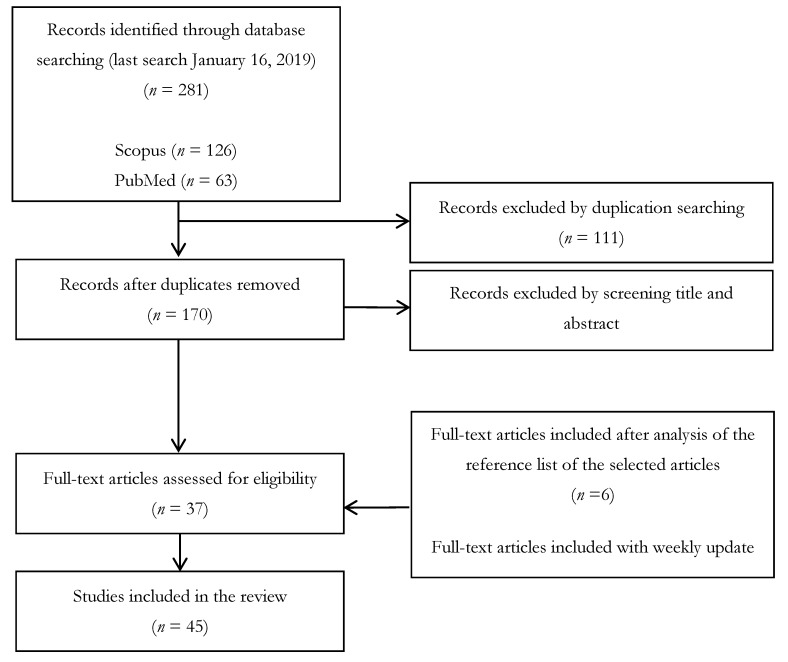
Flowchart of literature searched and reviewed, modified from Moher et al., 2009 [10].

As reported in Figure 1, a total of 126, 63, and 92 papers were found using Scopus, PubMed, and ISI Web of Knowledge, respectively. All papers (*N* = 281) were independently reviewed by two of the authors who selected the papers that were relevant for the review purposes in accordance with the inclusion criteria.

The inclusion criteria included original, peer-reviewed articles, published in English, and reporting the evaluation of reliability (in terms of accuracy, precision, robustness, validation, or external validation) of selected tools for the estimation of inhalable occupational exposure to chemicals. Exclusion criteria included case reports, conference papers, and publications that did not focus on occupational inhalation exposure (for this reason, both studies regarding dermal exposure or consumer exposure were not considered in this review), or that were published in languages other than English. Duplicates were removed from the total number of papers. Of the remaining 170 articles, two authors independently excluded 133 as they did not meet the inclusion criteria based on the title and abstract analyses. A total of 37 papers remained for review. The full texts of the articles that were considered suitable for review were obtained and subjected to a critical evaluation. By assessing the reference list accompanying the selected articles further enlarged the citation pool of relevant publications that were identified in the literature search; this allowed for the inclusion of 6 additional eligible papers. Weekly updates of the research in scientific databases were performed until the date of submission of the manuscript: this allowed for the inclusion of 2 additional eligible papers. Overall, 45 papers were retrieved and considered suitable for this review; the complete list is provided in Table S1.

A detailed flowchart of the literature is reported in Figure 1. The results of the eligible studies are described in the following sections and then organized into tables summarizing information concerning the kind of evaluation performed in the selected studies, considering different exposure scenarios (in terms of activity and chemicals), and outlining principal results regarding limits and advantages of different models.

## 3. Results and Discussion

### 3.1. Models Overview

The use of occupational exposure assessment models should follow a tiered approach: the evaluation should be organized into a first phase of evaluation (Tier1) in which simple tools should be used for exposure assessment. These tools should provide conservative and protective estimations (i.e., overestimated exposures) and be able to discriminate between an exposure scenario of concern and one that is not a threat. Following the REACH recommendation, in the event that the estimated exposure value is not controlled (i.e., the ratio between estimated exposure and the considered occupational exposure limit value is above the unit value), indicating that the potential presence of risk in the exposure scenario, or whenever some concern persists in a worker’s exposure to a chemical, then it is necessary to proceed with a second evaluation phase (Tier2), in which more advanced tools are used for exposure estimation (which should provide a more accurate and precise result than Tier1 tools), or environmental monitoring could be used to confirm the estimated exposure [1,11]. In more detail, Tier1 exposure models are characterized by a small number of input parameters and aim to provide conservatively (i.e., overestimated) modeled exposure estimates. These kinds of models, due to their simplicity and level of conservatism (which may also lead to a high level of uncertainty), are designed to easily identify situations that may pose a risk to health and are generally characterized by a broad range of applicability [7]. Tier2 models are more complex and detailed than Tier1 models and should not be considered as conservative as Tier1 tools. Tier2 models are more specific, and require detailed input parameters and a good characterization of the exposure scenario and the exposure determinants [2,9,12].

#### 3.1.1. Tier1 Models: ECETOC TRA, EMKG-Expo-Tool, and MEASE

ECETOC TRA is a generic model for both inhalation and dermal exposure of workers [13] and could be used without specific training, even if specific training is recommended [14]. ECETOC TRA is based on the descriptors used for processes categories (PROCs) defined under the REACH Regulation. Initial exposure estimates are derived from Estimation and Assessment of Substance Exposure (EASE) [15], but adapted to more recent exposure experience; the initial exposure estimates are subsequently modified based on a number of modifying factors [16]. The model only requires a few input parameters, but it covers many different scenarios. Exposure to aerosol and mist are not in the domain of ECETOC TRA and results regarding these scenarios should be carefully interpreted. Results obtained by ECETOC TRAv3 represent the 75th percentile of the exposure distribution [16], while ECETOC TRA v2 outputs are assumed to represent the 90th percentile [7]. The EMKG-Expo-Tool considers the whole mixture as a pure substance and does not consider the proportion of a substance in a mixture; the EMKG-Expo-Tool produces an exposure range as an output [7,17]. MEASE is derived from the ECETOC TRA tool (version 2) and it covers exposure to metals and inorganic substances; MEASE provides point estimates of exposure [6].

#### 3.1.2. Tier2 Models: STOFFENMANAGER^®^ and ART

STOFFENMANAGER^®^ is a web-based dermal and inhalation exposure model, initially developed to facilitate risk assessment of chemical in small- to medium-sized industries by means of control-banding [18]. For the purposes of this study, STOFFENMANAGER^®^ was considered among the Tier2 models, but the model is actually considered a Tier1.5 model (i.e., between a Tier1 model and a Tier2 model), since the model is easy to use and does not need detailed information and descriptors as input, but it is considered a more refined version of Tier 1 models [1,12]. The algorithm and general assumptions used in STOFFENMANAGER^®^ (versions 3.0–4.0) are based on the conceptual model developed and proposed by Cherrie and Schneider [19] and Marquart et al. [18], as described in Tielemans et al. [20]. Modifications to these earlier versions have been made which are listed on www.stoffenmanager.com (see under More, What is Stoffenmanager^®^). In the first step of the algorithm, a score is calculated as the sum of main exposure source near-field (NF; within 1 m of the worker’s head) and far-field (FF) and diffusive sources [21]. In the second step, a mixed-regression model based on calibration with experimental data is used to derive quantitative exposure estimates [22]. Results can be expressed as different percentiles of the exposure distribution: the 90th percentile outcomes are recommended for this model to ensure a conservative result [1].

ART is the most sophisticated and advanced tool for the evaluation of exposure levels under the REACH regulation, and for this reason it should only be used by expert assessors [14]. The model differentiates between different exposure processes: vapor, mist, and dust. This means that fumes, fibers, and gases are not considered by the model [23]. ART is based on a mechanistic model combined with an empirical component related to exposure databases [12]. ART (version 1.5) includes a Bayesian module that can be applied to the mechanistic model to adjust the estimated exposure [24]. In detail, the model is based on an algorithm that adopts a source receptor approach, describing the transport of contaminants from a source to a receptor, and considering different modifying factors (i.e., substance emission potential, activity emission potential, localized controls, segregation, personal enclosure, surface contamination, and dispersion). In the model’s algorithm, the workspace is ideally divided into different compartments: (1) NF (within 1 m of the worker’s head) and (2) FF (comprising the remainder of the workspace) [23]. The model provides estimates at different percentiles of exposure, within different confidence intervals. The 75th or the 90th percentile of estimates are recommended to be used as outcomes for this model.

#### 3.1.3. TREXMO

TREXMO is a tool that integrates different exposure models (ART v.1.5, STOFFENMANAGER^®^ v.4.0, ECETOC TRA v.3, MEASE v.1.02.01, EMKG-Expo-Tool, and EASE v.2.0) [5,25]. The tool is able to provide users with the most appropriate parameters to use in the other models in a given exposure situation, defined by a set of parameters in one of the mentioned models [5].

### 3.2. Methods for Model Performance Evaluation

Different analysis methods were used to evaluate model performance (in terms of precision and accuracy) in the studies considered in this review. A summary of those methods is reported in Table 2 and discussed afterward. The lack of agreement was calculated between model estimates and measured concentrations by different authors [22,26,27]. The precision of the models was calculated by Koppisch et al. [21], followed by Hornung [28], Schinkel et al. [22], and Lee et al. [27]. The aforementioned authors, Bekker et al. [29], and Heussen and Hollander [25] also reported results regarding the bias of models, considering absolute and relative differences between estimates and measurements or, in the case of Heussen and Hollander [25], between STOFFENMANAGER^®^ and TREXMO estimates. Regarding correlation and regression analysis, Bekker et al. [29] performed a correlation analysis between the natural logarithms of the ART estimates and measured concentrations, whereas Koppisch et al. [21] calculated the correlation between the measured 90th percentile and the GM (geometric mean) of the predicted 90th percentile per scenario. Schinkel [22] and Spinazzè et al. [2] performed regression and correlation analysis between estimated and observed exposure. Finally, the correlation and the consistency between exposure estimates from different models were calculated by Savic et al. [30]. In the same study, a multiple linear regression analysis was applied to determine how single determinants, such as vapor pressure, can affect differences the between models estimates [30]. A multiple linear model was also used by Lamb et al. [31]. Fransman et al. [23] used a mixed-effect regression model to evaluate differences between model scores and measurements, whereas Schinkel [32] conducted a logistic regression model to evaluate the accuracy of the assessment. Hesse et al. [12] and Ishii [33] calculated the ratio of the exposure estimate to the measurement value. Other authors [12,21,33] calculated the percentage of measurement exceeding the exposure estimates. In some studies [12,33], the authors compared the 75th and the 90th percentiles of estimates with measurement results. Finally, Landberg et al. [14] compared the Risk Characterization Ratios (RCRs) of selected Exposure Scenarios reported in extended Safety Data Sheets, with newly estimated scenarios’ RCRs. Landberg et al. [8] also evaluated the conservatism of the tool, comparing the modeled consensus with the measured median exposure. Some other statistical methods (uncertainty factor, evaluation of residual, Cohen κ statistics, and intraclass correlation coefficients) were applied in the reported studies. Spinazzè et al. [2] used the uncertainty factor in their evaluation; residuals were calculated as the log differences between estimates and measured exposure in another study [27]. Schinkel et al. [32] estimated the agreement between assessors using the Cohen κ statistic as the proportion of agreement beyond that expected by chance alone. Schinkel et al. [32] investigated the inter-assessor agreement using the intraclass correlation coefficient. Landberg et al. [8] evaluated the variability of multiple users’ outcomes. In the same study, the evaluation of the choices of input parameters from multiple users were compared with the modeled consensus (calculating the percentage of users’ agreement with consensus for each parameter considered) and the input parameters and their impacts on the outcomes were discussed. Finally, Koivisto et al. [34] reported the recalculation of the general ventilation multiplier (NF and FF multipliers) for STOFFENMANAGER^®^ and ART.

### 3.3. PROC, Chemicals, and Single Determinants Investigated

To identify which kind of exposure scenarios have already been considered for model evaluation studies, information was gathered regarding the evaluated exposure scenarios in terms of process categories, chemicals, and particular investigated determinants. REACH uses different descriptors to describe identified uses (e.g., sector of use (SU), process category (PROC), product category (PC), article category (AC) and environmental release category (ERC)). Many of those descriptors can be used as input parameters to derive exposure estimates in modeling tools. Following the definitions of PROCs provided by the ECHA, which describes the tasks, application techniques, of process types defined from the occupational perspective, including use and processing of articles by workers [1,35], all manuscripts considered in this review were examined in a subjective (when the PROC was not clearly reported in the paper) or in an objective manner (when the PROC was clearly stated in the paper), as reported in Table 3. The following PROCs (48.4% on total PROCs) have not been evaluated by any paper: PROC 6, 11, 12, 16–18, 20, 21, 23–26, 27a, 27b and 28. Similarly, 35.5% of PROCs were evaluated only once: PROC 1, 3, 5, 7, 8a, 8b, 9, 13–15, and 19. Of the PROCs, 9.6% were evaluated twice: PROC 2, 7, and 10; whereas PROCs evaluated by 3 different papers (6.4% of the total) included PROC 4 and 22. As expected, most papers (*N* = 29) evaluated PROC 0 (others), mostly due to problems assigning poorly described activities to a PROC by the authors.

Table 4 summarizes the models’ evaluation regarding chemicals used in the evaluated exposure scenarios. Van Tongeren et al. [7] considered a wide range of substances (powders, metals, non-volatile liquids, and volatile substances). General powder and dust scenarios were considered by Landberg et al. [26], Savic [44,45], and Schinkel [22,32,46], and scenarios concerning solids were evaluated in other studies [22,30,44,45]. Activities involving nano-powders (particularly TiO_2_, Al_2_O_3_, and SiO_2_) were investigated by Bekker et al. [29] and Liguori et al. [47]. Liquid scenarios were considered [26,32,44], as were vapors and mists [30,45,46] and volatile substances [7,22,27,30,43]. Other chemicals were evaluated by Landberg et al. [14], and others evaluated benzene [24], ethylbenzene [33], toluene [3,37,42], ethyl acetate [36,39], and acetone [36]. Petroleum substances were investigated by Hesse et al. [12], including kerosene, heavy fuel oils, the naphtha-2 group, gas oils, and other lubricant base oils. Solvents-related exposure scenarios were investigated by Spinazzè et al. [2], Zaleski et al. [48], and Lee et al. [27]. Copper pyrithione [39], welding fumes [49], 1-methoxypropan-2-ol [13], co-formulants used in plant protection products [50], consumer spray product [51], pesticides [36], inorganic complex fertilizer [52], polyurethane foam [42], and Sevoflurane [38] were evaluated in different studies.

Regarding the evaluation of the effects of specific determinants or modifying factors (MF) on estimates results, only seven determinants were deeply evaluated in the studies considered in this review (Table 5). McDonnell et al. [41] described scenarios using the main MFs: (1) activity emission potential, (2) substance emission potential (categories grouped to dust or granules), and (3) localized controls. Koivisto et al. [34] conducted extensive work on general ventilation multipliers, whereas Park et al. [51] evaluated ventilation rate, the room size, and the amount of aerosol sprayed. Two studies completed a sensitivity analysis to investigate MFs’ impacts on estimation results [2,3].

### 3.4. Model Performance

#### 3.4.1. Tier1 Models

##### ECETOC TRA

Regarding the ECETOC TRA model, 14 scientific articles (Table 6) were found to be suitable for inclusion in this review and then further analyzed. The analysis results about the ECETOC TRA showed that different authors described the model as not conservative enough to be a Tier1 model in several exposure scenarios, especially if compared with other tools.

Spinazzè et al. [2] outlined that although results obtained with ECETOC TRA v3.1 generally showed a good level of conservatism, ECETOC TRA cannot be considered acceptable in terms of accuracy. In detail, the study found that ECETOC TRA provides: (1) unrealistic (but highly conservative) prediction considering pesticide application, suggesting that the model is not appropriate for an accurate evaluation of this kind of chemical (and, in general, chemicals with extremely low volatility used in spray applications); and (2) acceptable estimates for solvent-related scenarios (but which show an insufficient level of conservatism in some cases, anyway). The authors concluded that, overall, ECETOC TRA v3.1 could be used as a first screening tool for inhalation exposure scenarios. Van Tongeren et al. [7] evaluated different lower tier models, including ECETOC TRA (versions 2 and 3), reporting results of the external validation of estimates using measured data. The authors tested the performance of the models across a wide range of exposure scenarios and substances, including volatile substances, powders, metals, and non-volatile liquids. The performance of ECETOC TRA was found to be different for different chemicals. Estimates were less conservative for volatile liquids (liquids with vapor pressure >10 KPa at room temperature). Similarly, a lower level of conservatism was found for highly dusty material in powder handling, whereas a medium–low level of conservativism was found in metal abrasion exposure scenarios. ECETOC TRA was not considered sufficiently conservative for specific chemicals (volatile liquids) in the ETEAM (Evaluation of Tier 1 Exposure Assessment Models) project [9] and by Hesse et al. [12], where the authors showed how the model was unable to consider all possibilities for the heavier, less volatile, and more complex petroleum substances of those included in their studies. As reported in Lee et al. [17], the ECETOC TRA v.3 is not sufficiently conservative for the selected exposure categories reported in their study. ECETOC TRA is described in Landberg et al. [54] as not conservative enough to be a Tier1 model due to the high risk of accepting false safe scenarios (i.e., a situation in which risk assessment based on models were deemed safe, but measurements deemed the situation unsafe). Specifically, the risk of false safe exposure estimates is higher when using ECETOC TRA than other models used in the study (i.e., STOFFENMANAGER^®^ and ART). In another study, Landberg et al. [14] evaluated the modeled outcomes compared with chemical exposure measurements. The results showed that when the default outcome of ECETOC TRA was used, 31% of the measured exposure exceeded the modeled exposure. Compared with other models used in this study (i.e., STOFFENMANAGER^®^ and ART), ECETOC TRA was the least conservative. The poor performance of ECETOC TRA was reported by Lee et al. [27]. They evaluated the performance in terms of accuracy, precision, and conservatism of three exposure tools, including ECETOC TRA v.3.1, during solvent cleaning tasks. When compared with STOFFENMANAGER^®^ and ART, ECETOC TRA produced less accurate outcomes, with a lower level of conservatism, and weaker correlations, and the authors observed a systematic tendency to overestimate low exposures and to underestimate higher exposure situations. ECETOC TRA has been evaluated across different scenarios, as reported by Jankowska et al. [38]. The authors assessed the potential use of exposure tools to estimate professional exposure to chemicals in particular workplaces (exposure to sevoflurane in operating rooms). The results showed that ECETOC TRA tends to overestimate the concentration approximately 20-fold compared with measurement data. Kupczewska-Dobecka et al. [42] aimed to develop a strategy for the assessment of exposure to isocyanates (TDI: a mixture of toluene-2,4- or 2,6-diisocyanate; MDI: methylene bisphenyl isocyanate) during production of polyurethane foam. In this study, ECETOC TRA seems to be adequate as a Tier1 model in this peculiar scenario since the model estimated the concentrations values in a conservative manner, supporting the need to carefully choose the most representative process category (for this study, PROC12) to obtain an optimal result. Vink et al. [13] reported results regarding a tiered exposure assessment for the risk characterization of 1-methoxypropan-2-ol (PGME) using different exposure tools (including ECETOC TRA v.2). As expected, high variability was reported in estimate outcomes from lower tier models such as ECETOC TRA; the highest inhalation exposure estimates were obtained with ECETOC TRA, which was also found to be sufficiently conservative (the measured inhalation exposure levels were generally below those estimated with ECETOC TRA, but above those obtained with STOFFENMANAGER^®^ v.4). Spee and Huizer [11] studied the exposure to methyl methacrylate (MMA) during the application of polymethylmethacrylate (PMMA) in floor coating. The results showed that in 86% of the cases, the measured exposure was higher than the estimates calculated using ECETOC TRA and, even when recalculating the estimation using a more realistic ventilation parameter, the results were unchanged. The authors of the study underlined that the temperature may be a determinant influencing the performance of the ECETOC TRA model. A sensitivity analysis of factors most influencing the model was performed by Riedmann et al. [3]. The authors found that the single most important factor affecting the model is the selection of PROC (24% for solids and 30% for liquids). This means that, as reported by the authors, a failure to identify the PROC might severely influence the related exposure estimates. Regarding reported modifiers of the model [12], the ECETOC TRA v.2 algorithm offers a basic set of modifiers (for both operational conditions and protective measures) but they were not considered by the authors as sufficient for the description of typical petroleum-related activities (handling and application of petroleum products). Tischer et al. [9] presented the ETEAM project overview and the methods used. The ETEAM project aimed to assess the between-user reliability of different exposure assessment tools, including the ECETOC TRA model (versions 2 and 3) and, for this reason, detailed information regarding the outcomes of this study is reported in Section 3.5. Savic et al. [30] evaluated different model performance using TREXMO (better discussed in Section 3.4.3). In this work, the authors evaluated correlations between the exposure estimates calculated by pairs of models, including ECETOC TRA. Concerning ECETOC TRA, the results generally showed that the model is characterized by acceptable performance. However, results of this study also suggested that the tiered approach seems to not be generally applicable to all exposure situations analyzed in the study. On this basis, authors then suggested the need for a multiple-model approach to critically assess exposure scenarios under REACH and outlined the need for further occupational studies to improve the prediction accuracy of the models in general [30]. Ishii et al. [33] evaluated ECETOC TRA’s performance for investigating 137 tasks related to manufacturing and painting in 17 companies; the authors reported that the model can be adequately used as a Tier1 model for screening assessments in the investigated contexts. Angelini et al. [43] used ECETOC TRA v.2 to evaluate occupational exposure to solvents in different workplaces, and compared estimations with measured exposure data. The results showed that only 37% of the values obtained with the ECETOC-TRA method were above experimental values. Hofstetter et al. [37] concluded that ECETOC TRA overestimated the concentration in the occupational setting considered (toluene in the spray scenario) by a factor of 3.61, thus providing an adequate level of conservatism. The authors also determined that the model provides relatively precise and conservative estimates according to the level of detail of the model.

##### MEASE

Regarding the MEASE tool, only three papers (summarized in Table 7) were found to be suitable for inclusion in this review. Lamb et al. [31] investigated the between-user reliability of different Tier1 tools, characterizing differences in the choice of input parameters between users. They also considered the MEASE tool but, because of its design, the results of this work are better reported in Section 3.5. Tischer et al. [9] principally examined the between-user reliability, and their results are reported in Section 3.5. Van Tongeren et al. [7] found that estimates obtained with MEASE were higher or similar to exposure data. In detail, during powder handling tasks, the tool was found to be less conservative for medium dustiness, whereas a medium level of conservativism was found for metal abrasion and processing. MEASE was found to be insufficiently conservative for exposure to non-volatile liquids.

##### EMKG-EXPO Tool

Five papers concerning the EMKG-Expo-Tool model were included in our review (Table 8). Lamb et al. [31] investigated the between-user reliability in the Tier1 exposure tool, but some outcomes regarding the EMKG-Expo-Tool can be extrapolated from their work. The authors stated that the model includes a scale of use factor, but that the percentage of the agent in the mixture is not considered by the model. The authors proposed that this lack can cause a difference in estimates between EMKG-Expo-Tool and other exposure tools. Lee et al. [17] evaluated lower tier models and found the EMKG-Expo-Tool highly conservative for the considered chemicals, except for liquids with high vapor pression; the model was found to be the most conservative among the others considered in this study (i.e., ECETOC TRA and MEASE). Regarding volatile liquids, Van Tongeren et al. [7] reported evaluation results divided as a function of the substance considered. For volatile liquids, EMKG-Expo-Tool was the only tool (among ECETOC TRA, MEASE and STOFFENMANAGER^®^) that was found to be highly conservative for these types of chemicals. Spee and Huizer [11] evaluated the exposure to methylacrylate during the application of polymethylmethacrylate floor coating; the estimates performed via EMKG-Expo-Tool resulted in an unsafe exposure situation in all scenarios considered, in comparison with the measured exposure. A summary of the ETEAM project [9] is reported in Section 3.5.

#### 3.4.2. Tier 2

##### STOFFENMANAGER^®^

Regarding STOFFENMANAGER^®^, a total of 21 papers were found suitable for inclusion in this review. Detailed information is provided below and in Table 9.

Spinazzè et al. [2] evaluated the accuracy and robustness of different exposure models, including STOFFENMANAGER^®^, comparing available measurement data of exposure to organic solvents and pesticides with model estimates. In general, the authors found that STOFFENMANAGER^®^ was the most robust model used in the study (among ECETOC TRA and ART). This model can be considered a safe alternative to other tools used, especially when detailed information is difficult to assess. The authors declared that STOFFENMANAGER^®^ appears to be the best model among those considered for the estimation of low-volatile substances and for evaluation of pesticide application. The balanced feature of the model was also underlined by Lee et al. [27]. The authors evaluated the accuracy, precision, and conservatism of three different occupational exposure models, including STOFFENMANAGER^®^, comparing model prediction and measurements during solvent cleaning tasks. STOFFENMANAGER^®^ was also found to be the most balanced model (good accuracy, high correlation, medium conservatism) amongst the other tools considered. The authors observed a tendency of the model to overestimate low exposure and to underestimate higher exposure in all considered models.

Similar results were reported by Landberg et al. [26]. The authors investigated the validity of the model, comparing the lack of agreement between estimates and measured exposure in seven different industries concerning the handling of liquids and powders. In general, the authors found that in the investigated scenarios, the model tends to overestimate situations characterized by low exposure and underestimated those with high measured exposure. Landberg et al. [26] reported that the model in general has a higher agreement in estimated vs. measured concentration in powder handling scenarios besides situations with liquids handling. In another study, Landberg et al. [14] evaluated the modeled recommended outcomes (namely, the conservative choice) and compared the outcomes with the measured exposure, evaluating the level of conservatism. In the evaluated scenarios, when the 90th percentile was used, 17% of the measured exposure was higher than modeled, even if the results were close to each other, which means that the modeled exposure was close to the measured exposure. Landberg et al. [14] evaluated the risk assessment approach of the REACH legislation in industrial setting evaluation and in comparing RCRs. The most false-safe scenarios were detected using STOFFENMANAGER^®^ in this study compared with ART and ECETOC TRA. Van Tongeren et al. [7] described the results of external validation of different exposure models (including STOFFENMANAGER^®^) using measured data. Regarding volatile liquids, the authors found that the prediction outcomes from the tool agreed with measurement results, which were more conservative at higher exposure levels. STOFFENMANAGER^®^ seems to underestimate exposure during the use of non-volatile liquids while providing highly conservative predictions for powder exposure. More specifically, for STOFFENMANAGER^®^, available results for nonvolatile liquids related to PROC 11 (non-industrial spraying activities) suggested that STOFFENMANAGER^®^ was not sufficiently conservative for non-volatile liquids when applied within this PROC. Furthermore, for volatile liquids, STOFFENMANAGER^®^ underestimated the exposure compared to the measurement results for PROC 14. For powder handling, STOFFENMANAGER^®^ was highly conservative for PROCs 5, 7, 8b, 9 and 14 (as for ECETOC TRA and MEASE). Finally, STOFFENMANAGER was less conservative for PROC 8a, which relates to less controlled powder transfer processes at nondedicated facilities. Moreover, when assessing exposure to low volatile substances, released from spraying activities (i.e., PROC7 and PROC11), results could be possibly underestimated. To overcome this issue, for such activities STOFFENMANAGER^®^ developers recommended using the 95th percentile estimation, or to comply with an RCR = 0.5 instead of RCR = 1 (www.stoffenmanager.com-More, What is Stoffenmanager^®^). Lee et al. [53] evaluated STOFFENMANAGER^®^ and ART in terms of accuracy and robustness for 19 different workplaces. The results also showed that the model appears to be reasonably accurate and robust for estimates of liquids with VP >10 Pa, even if some improvement could be useful. Ribalta et al. [40] explored the applicability of different methods to determine the statistical significance of coarse particle emission during activities related to the packing of ceramic materials. Results of the STOFFENMANAGER^®^ performance showed that the model tends to overestimate concentrations by factors between 1.6 and 2.9. A cross-validation study was also performed [22] comparing exposure estimates with exposure measurements. Analyses were performed for different scenarios (handling of powder and granules, handling solids resulting in commuting, and handling of low-volatile and volatile liquids). Results of the cross-validation confirmed that STOFFENMANAGER^®^ can be used as a Tier1 model for regulatory risk assessment, because even the 90th percentile estimates of the model were found to be sufficiently conservative. A sensitivity analysis of different occupational exposure models was performed by Riedmann et al. [3] with the aim of determining which factors most influence the estimate results. The authors found that in STOFFENMANAGER^®^, the maximum difference between the most and the least important determinants varies by a factor of four (vapor) and by a factor of three (mist and dusts). The model was found to be the most balanced tool between among those considered. Koivisto et al. [34] revised calculations regarding multipliers used by different Tier2 models (STOFFENMANAGER^®^ and ART). The authors, following Cherrie’s [55] procedure, calculated NF and FF concentration ratios. The results showed that the recalculated general ventilation multipliers with respect to STOFFENMANAGER^®^ evaluations were up to 2.8 times than the values reported in Cherrie’s [55] study. The recommendations provided by the authors included multipliers and the error associated with the general ventilation multipliers, which may require revision. Koppisch et al. evaluated and explored the usefulness of the MEGA database for validating STOFFENMANAGER^®^, in particular, for equations derived from Schinkel et al. [22] for estimating the occupational exposure to inhalable dust. The authors emphasized the need for uniform data collection and storage. The MEGA database was used to extrapolate information regarding select scenarios, and the utility of this tool for the model validation process was confirmed, even if some further implementation was recommended. The authors explained that if this kind of database and tools is more frequently used in the future, it will be necessary to acquire and store information centrally in compliance with specific requirements. Finally, other authors used STOFFENMANAGER^®^ for particular and detailed scenarios: Vink et al. [13] illustrated critical elements in a non-testing approach, specifically during professional painting activities. The authors reported a large variability in the different models considered (including STOFFENMANAGER^®^) in comparison with measured data. A specific scenario was evaluated by Ribalta et al. [52]. The authors performed a worker exposure and risk assessment study of packaging of an inorganic complex fertilizer. In this scenario, the tool tended to overestimate the exposure level, with some exceptions where estimates results were accurate. Other scenarios were evaluated, such as in operating rooms with use of sevoflurane as the anesthetic gas [38]. In these kinds of scenarios, STOFFENMANAGER^®^ provides accurate estimates and can definitively be used as a screening tool for the assessment of occupational exposure to these kinds of chemicals. STOFFENMANAGER^®^ (and exposure models) can also be used to evaluate intervention studies, such as reported in Terwoert et al. [56]; in this study, authors discussed how the use of validated tools embedded in a community platform, supported by active training and coaching, helped companies to improve their chemical risk management, to avoid making mistakes when using and applying STOFFENMANAGER^®^ and to organize and structure their chemical risk management policy. Some studies [8,9,31] aimed to assess the between-user reliability of the model. In particular, Landberg et al. [8] aimed to (1) investigate multipliers used in STOFFENMANAGER^®^ algorithms and (2) to evaluate the conservatism of the model. The authors investigated and reported airborne exposure across different scenarios (metal foundry, wood, printing, and spray-painting industry), calculated by different users.

As already mentioned, Lamb et al. [31] investigated the between-user reliability of different models; Tischer et al., with the ETEAM project [9], aimed to evaluate Tier1 exposure assessment models, including STOFFENMANAGER^®^, with regards to the between-user reliability of exposure assessment tools. Further details of the studies that specifically examined the between-user reliability are reported in Section 3.5. Section 3.4.3. discussed studies concerning STOFFENMANAGER^®^, but performed within the framework of investigation of the TREXMO tool [25,30,44].

##### ART

Regarding ART, a total of 24 papers were found suitable for inclusion in this review. The information about these papers is detailed below and listed in Table 10. The details of model construction and the general outline was also reported.

As reported by the authors, the combination of the ART model and the ART exposure database enables users to estimate occupational exposure using a state-of-the-art approach. Fransman et al. [23] described the development of the ART model and characterized the modifying factor used in the model algorithm. The authors report that because each assigned multiplier of a modifying factor is characterized by a natural variability around a median value, future versions of the model might add distribution for the multipliers for each modifying factor. Tielemans et al. [57] reported detailed information regarding the general outline of ART (version 1.0), including the mechanistic model, exposure predictions, and applicability domain of the model. Technical details of the ART model were also reported by McNally et al. [39]. A calibration of ART was performed by Schinkel et al. [46], who studied whether the mechanistic model scores are accurately ranked in relation to exposure measurement and provided a method for quantifying model uncertainty. The mechanistic model was found to be able to estimate GM exposure (90% confidence) of a scenario. Schinkel et al. [58] described the structure functionalities and content of the ART exposure database. Spinazzè et al. [2] evaluated the accuracy and the robustness of different exposure models (STOFFENMANAGER^®^, ECETOC TRA, and ART), comparing measured data in occupational exposure scenarios involving the use of organic solvent and pesticides. In this study, ART was found to be the most accurate model among others, even if the model tended to underestimate exposure to pesticides. Conversely, ART was the most accurate regarding organic solvent exposure scenarios. Similar results were reported by Lee et al. [27] who evaluated the performance of exposure models (ECETOC TRA, STOFFENMANAGER^®^, and ART), comparing model estimates and exposure measurements for solvent cleaning tasks. In this study, ART was also found to be the most accurate and precise model among the others chosen in this study, even if the conservatism of the model was classified as medium. The authors observed a systematic tendency of the model to overestimate lower exposure and to underestimate higher exposure in all considered models. This tendency was confirmed by other studies. Bekker et al. [29] evaluated model performance in scenarios involving dumping and mixing of nano-powders (TiO_2_, Al_2_O_3_, and SiO_2_). The results showed that the model overestimates exposure at low concentrations, which decreases with the increase in concentration. The authors also evaluated the effects of various determinants on model output. The results showed a moderate–strong correlation between estimates and measured concentrations, even if estimates correlated better for dust than for liquid aerosol. Hofstetter et al. [37] evaluated the performance of the ART model in occupational scenarios. As expected, the model estimates aligned with experimental results, thus proving its suitability as a Tier2 model. ART’s estimates were also compared with exposure measurements by Savic et al. [45]. The exposure at the 50th and 90th percentile outcomes from a Swiss database concerning exposure to vapor, mist, powder, and abrasive dust were calculated in ART. The results showed that ART’s performance at the 50th percentile was insufficiently conservative with regard to exposure to wood/stone dust (abrasive dusts), whereas the 90th percentile showed sufficient conservatism for all types of exposure examined. ART tended to overestimate lower exposure and underestimate high exposure levels. The authors suggested using the upper level of the 90% CI of the 90th percentile for predictions involving vapor and powder and to use the upper level of the 95% CI in the 95th percentile for scenarios involving solids. The outcomes from the study conducted by Ribalta et al. [40] showed how the model overestimates exposure concentration in the evaluated scenario (packing of ceramic material), although the ART mechanistic model tended to underestimate concentrations in some cases. Landberg et al. [26] investigated the validity of exposure models (in particular STOFFENMANAGER^®^, and ART), comparing the lack of agreement between modeling tools and measured exposure during handling of liquids and powders. The authors found that ART tends to underestimate the exposure and agree less for specific activities (wood industry), whereas activities involving handling liquids agreed more than in situations concerning the handling of powders. In another study, Landberg et al. [14] investigated different risk assessment approaches for exposure to chemicals in seven kinds of industries (wood, printing, foundry, spray painting, flour milling, chemical, and plastic molding). All exposure situations were assessed with ART and with the Bayesian algorithm in ART (ART B). The results showed that when the upper 95% CI of the 90th percentile outcomes from ART were used, the measured exposure exceeded the estimates in only 3% of the cases considered (one situation). When considering the same output but ART B, none of the measured exposures exceeded the estimates. Landberg et al. [14] aimed to evaluate the risk assessment approach of the REACH legislation across different industrial chemical departments. In this case, Landberg et al. declared that ART (and exposure models in general) as well as generic ES (exposure scenario) should be used with caution when chemicals are characterized by a high vapor pression and low DNELs (Derived No Effect Level). Similarly, Lee et al. [53] evaluated higher-tier models (STOFFENMANAGER^®^ and ART) in terms of accuracy and robustness. The results showed that ART’s median prediction seems to be reasonably accurate for liquids with a vapor pressure >10 Pa. The model underestimated exposure for all different tasks considered except for activities with relatively undistributed surfaces. Another study [41] aimed to refine and validate the inhalable dust algorithm of the ART model to predict airborne exposure in the pharmaceutical industry. The results showed that in 12 of the 16 scenarios investigated, GM exposure estimates were lower than the measured exposure level (characterized by a relative bias of −32%). As reported by the authors, the general uncertainty of the model is due to a combination of model and parameter uncertainty. In another analysis performed with the aim of understanding influencing factors, LeBlanc et al. [24] evaluated benzene exposure during the use of a metal parts washer that were modeled using ART, also applying Bayesian analysis. As expected, the application of this implementation narrowed the confidence interval estimates in the exposure estimates, reducing the associated error. Riedmann et al. [3] performed a sensitivity analysis on different exposure models with the aim of determining the main factors that influence the models. The results showed that the background can be neglected and that the most important and influential factors are local controls and source emission. Koivisto et al. [34] revised the calculations that produce multipliers both in ART and STOFFENMANAGER^®^. The results showed that the recalculated general ventilation multipliers were up to 2.8 times greater than the values reported in Cherrie’s study [55]. These results suggest that the error in general ventilation multipliers may be relevant and, for this reason, the authors suggested revising multipliers for general ventilation in ART and STOFFENMANAGER^®^. Van Tongeren et al. [59] described deviations from factors that are used to model releasers of dust, mist, and vapors. The study showed that for handling powders, granules, and pellets, the qualitative assignment of the dustiness category of a product seems to be appropriate.

Regarding the handling of solid objects, the authors reported that the characteristics of solid materials (structure, friability, and hardness) are considered important factors in the mass and the particle size distribution of the aerosol emitted during abrasive activities. The evaporation was found to be the main process emitting volatile liquids, influenced by the rate of evaporation (depending on the volatility of the liquid, the surface area of the source, and by environmental conditions such as air temperature, velocity, direction, and turbulence). Finally, regarding the handling of low-volatility liquids resulting in exposure to mist, the authors reported how mist can be generated by different processes, such as impaction of a liquid on a surface, the bubbling of gases through a liquid, or by evaporation. Finally, Sailabaht et al. [49] discussed the modifying factors that should be considered for inclusion in the model for welding fumes exposure and suggested acquiring more detailed information about the process to facilitate the use of data in exposure model development.

The reliability of the ART exposure model was evaluated in terms of inter-assessor agreement [32] and the findings are reported in Section 3.5. Section 3.4. reports the findings of Savic et al. [30] who evaluated different exposure models (ART, STOFFENMANAGER^®^ and ECETOC TRA) via comparison with TREXMO.

#### 3.4.3. TREXMO

Four papers about the TREXMO tool were found to be suitable for this review (Table 11). Savic et al. [5] described the development, validation, and performance of Translation Exposure Models (TREXMO) that are able to integrate six different exposure models. In their paper they mention integrating Stoffenmanager^®^ version 5.1 in TREXMO. This is later corrected in the tool itself to Stoffenmanager^®^ version 4.0 [25]. The aim of this tool is to produce a single user-friendly interface, helping users to select the appropriate parameters and to use different exposure models for the evaluation of a single scenario. In this work, the translation efficiency (number of possible translations of a parameter/set of parameters from one model to another) was calculated for every model using the TREXMO tool, using all defined exposure tools and groups of determinants. The results showed that this tool reduces the number of available parameters and the total number of combinations of parameters possible for each considered model. Starting from ART exposure scenarios and considering solids and dust, the source group determinants (i.e., dustiness, moisture content, and weight fraction) can define all the parameters in the other models and therefore could be directly translated. Liquid exposure scenario details (vapor pressure, activity coefficient, and weight fraction) allowed straightforward translation and no other choices were required. The authors stated how TREXMO can improve the between-user reliability, reducing the number of choices that single users must make. Savic at al. [30] also evaluated different exposure models-ART, STOFFENMANAGER^®^ and ECETOC TRA-via correlation and consistency analysis performed with TREXMO. In this study, the best correlation was found for the STOFFENMANAGER^®^–ART comparison even though the consistency varied significantly according to different exposure scenarios or settings. Despite this, the model was more consistent for (1) vapor than for dust and solids scenarios, (2) NF than FF, and (3) indoor than outdoor situations. Heussen and Hollander in their letter to the editor [25] calculated the absolute and relative differences between STOFFENMANAGER^®^ (considered the golden standard) and TREXMO in the context of a small random test. The results showed that a reduced number (1 of 20) of scenarios produced the same outcome in both tools, due to the use of different Stoffenmanager® versions. The authors concluded that TREXMO is not able improve the between-user-reliability, as expected. Savic et al. [44] performed a comparison between STOFFENMANAGER^®^, and TREXMO. The differences between TREXMO and STOFFENMANAGER^®^ (version 6) and the published model algorithm were investigated. Differences between the estimates calculated in TREXMO and estimates calculated manually were found to be insignificant. Savic et al. [60] reported that TREXMO improves the between-user reliability (Section 3.5). For TREXMO, sources of uncertainty related to the kind of scenario include (1) approximations of workplace floor and room volume and (2) unreported risk management measures such as local ventilation. Sources of uncertainty related to the model’s parameters are related to (1) data quality or (2) the subjective definition of some parameters (such as the dustiness of powder). Notably, the uncertainties reported above may further affect the overall model performances and should be reduced or limited [5].

### 3.5. Between-User Reliability

During the modeling phase of the exposure assessment, an assessor must interpret and translate an actual exposure scenario into model parameters and describe the exposure using the same range of determinants [31]. Exposure determinants can be described directly, choosing between more options given by the model or, in the case of a limited number of input choices being available, using the user’s experience. This means that a certain level of subjectivity is present and must be considered in all assessment process.

Lamb et al. [31] investigated the between-user reliability of Tier1 models within the Between-User Reliability Exercise-BURE. The authors found that the variance between tool users is not smaller in self-assisted experienced exposure, meaning that the user’s experience does not guarantee increased reliability. The major effects of participant characteristics (such as English language ability or increase in years of experience) have not been reported in estimates. Other results showed that participants usually report major uncertainty in selecting and allocating parameters referring to task/activity and not to other scenario parameters (substance characteristics, operational conditions, task/activity description, and risk management measures). Users also reported more uncertainty in the allocation of substance characteristics of solids compared with liquids. Given these results, the authors underlined the need of a training and implementation of additional controls and quality control system in all exposure models.

Schinkel et al. [32] aimed to assess the reliability of the ART exposure assessment model by studying and analyzing the inter-assessor agreement. In particular, the level of agreement between different assessors was evaluated by estimating the percentage of rating per exposure parameter. The intraclass correlation coefficient was calculated for exposure estimates derived before and after assessor training, and the absolute ration between the estimates calculated by different assessors and gold standard estimates was considered to understand the accuracy of exposure estimates. The results showed that a substantial variability was observed among estimates by different assessors. The reliability of estimates seems to be influenced by different factors: (1) information provided by text and video, (2) the implementation of guidance documentation being insufficient, and (3) the assessors being unable to implement the information explicitly provided.

Multiple users of the STOFFENMANAGER^®^ exposure tool were investigated by Landberg et al. [8]. The author found users struggled to assess four parameters having a large impact on the model results: type of task, breathing zone, personal protection, and control measures.

Another study [9], aimed at assessing the between-user reliability of exposure assessment models, and evaluated the consistency of users when making input parameter choices in the same situations. In this case, the authors underlined that assessments of similar exposure scenarios can vary considerably between users. Given this between-user variation and model uncertainty, higher confidence levels of conservatism may be necessary.

Savic et al. [60] also evaluated the inter-assessor agreement in different exposure scenarios using TREXMO. In this study, the assessors were asked to code given parameters, evaluating the exposure assessment using different methods. In more than half of the evaluated cases, the results showed better agreement between assessors selecting the exposure parameters within the framework of TREXMO than when manually coding. The most affected parameters were those related to activity (such as handling types in STOFFENMANAGER^®^) and exposure control (such as local controls).

It must be noted, however, that TREXMO uses earlier STOFFENMANAGER^®^ 4.0 algorithms which (partly) differ from the most recent algorithms of the original STOFFENMANAGER^®^ tool available at www.stoffenmanager.com (which also includes guidance, tooltips and descriptions). Thus, the conclusion, that the agreement between the estimates calculated by different assessors improved when performing translations between the models, could be considered premature and should be validated [25,44]. In summary, implementation of additional support and quality control systems for all tool users is needed to reduce between-assessor variation; inconsistency between tool users may generate estimates that differ by several orders of magnitude (as function of scenario, chemicals, and tool). There is thus a considerable probability of generating false negatives (i.e., where the scenario is assessed as safe, but where actual exposure exceeds the threshold value), or false positives [31].

### 3.6. Future Recommendations and Further Developments of Exposure Models

Continuous development, adjustment, and recalibration of the modeling tools are essential [4]. From the analysis of the available literature of the reliability of the occupational exposure estimation models, some issues emerged regarding the need for improvements of the models, to improve performance, or extend their domain of applicability.

Angelini et al. [43] proposed an improved version of ECETOC TRA (v.2) by adjusting four correction factors to integrate some exposure determinants (i.e., exposure duration, percentage of the substance in the composition, presence of collective protective equipment, and wearing of personal protective equipment). The validity of the improved model was verified using experimental values measured under real conditions in various exposure scenarios concerning handling (weighing mixing, packaging, and reconditioning-transferring) of organic solvents. The results outlined that 98% of the values obtained with the proposed improved model were above the experimental values measured in real conditions (while the classical version of ECETOC-TRA generated only 37% overestimated values), thus indicating a good level of conservatism. Concerning ECETOC TRA, some PROC were recommended for consideration in future tool upgrades or development (i.e., PROC 10 and 15) given low levels of conservatism. In addition, the algorithms for liquids with high and medium VP, profession and industrial domains, and situations without LEV (local exhaust ventilation) should be re-evaluated [17]. The EMKG-Expo-Tool estimates for high volatile liquids should also be re-evaluated [17].

Koivisto et al. suggested revising multipliers for general ventilation in ART and STOFFENMANAGER^®^ [34]. Concerning the need to extend the models’ domains to other chemicals, Sailabaht et al. proposed a change in the modifying factors of ART to include welding fumes exposure in the model’s domain [49].

Bekker et al. [29] outlined that, although ART is not capable of estimating occupational exposure to nano-objects and their aggregates and agglomerates (NOAA), ART and other generic exposure models have the potential to be extended or adapted for exposure to NOAA. Thus, the authors suggested that future development or refinement of NOAA exposure models should focus on investigating the effect of specific material characteristics on the dustiness of nanopowders and NOAA. Expansion of the ART methodology to include dermal exposure was also deemed important [57] and was recently proposed [61], as well as the integration of ART predictions of inhalation and dermal exposure with approaches for modeling internal dose [57].

Fransman et al. [23], while describing the development of the ART mechanistic model, outlined a number of issues concerning the characterization of modifying factors, and suggested that future versions of the mechanistic model might be improved by adding distributions for multipliers for each MF (as each MF has a natural variability). The authors identified other areas requiring further research, including the intrinsic emission potential of various solid objects, outdoor dispersion, and extending the applicability domain to other types of exposure (e.g., fumes, gases, fibers, etc.). The need to produce more validity studies was also reported [32].

In this regard, the availability of exposure measurements was recognized as a priority to continue validating and refining existing exposure models to increase the knowledge of exposure variability and the effect of exposure determinants on exposure levels [4]. The need for uniform data collection and storage has also been recognized for the construction of exposure databases to be expanded in the process of model development [21]. The need for a multiple-model approach was also suggested to improve the prediction accuracy of models in general when used in combination with occupational exposure measurements [30]. More generally, harmonization and calibration of the input and determinant parameters (e.g., room size, ventilation exchange rate, activity duration, activity energy, dustiness, and humidity influence) and the output are needed to improve the coherency and comparability of the results. Lastly, the improvements in guidance documentation, consensus procedures, training methods, and quality control systems could improve the reliability and reduce between-assessor variation [31,32,56]. In this context it is expected that a fundamental role in the harmonization, improvement and evaluation of existing models, as well as the development of new tools based on the latest techniques and knowledge, will be covered by networks of researchers within scientific societies, possibly with the participation of company parties and institutions.

## 4. Conclusions

Several studies have been conducted on the exposure tools used under REACH, but overall, little is known about the actual performance of these different models and their relative domain of validity, as well as for other models defined outside the REACH framework, which have not been considered in this study. A priori selecting which model is the most adequate is therefore challenging. Thus, to define the currently available evidence about the reliability of inhalation occupational exposure estimation models, a systematic review was performed. A total of 45 papers were retrieved and considered suitable for this review.

The results outlined that different analysis methods have been used to evaluate model performance, considering different reliability indicators (e.g., conservatism, robustness, precision, accuracy, uncertainty, comparison with measurement data, etc.), and most of the results refer to small-scale studies. This complicates the comparison between different studies, and further complicates the extension of the results obtained from one study to those of another, and the drawing of robust conclusions. Thus, we recommend performing a meta-analysis of existing evidence on model reliability to properly define the actual state of model performance. Future studies on this issue should be designed following harmonized procedures to improve the coherency and comparability of evaluations.

The available studies do not currently provide information about the reliability of the models for many of the main process categories coded under REACH. In many others, although information is available, the overview does not appear to be exhaustive, since, in most cases, the information was sourced from a limited number of small-scale studies, with inconsistent experimental designs. Only a few studies considered an adequate number of exposure scenarios/situations. Similarly, studies have focused on exposure to a limited number of chemicals or categories of chemicals, often without specific indications of the chemical agents considered. Few studies have examined the contribution of exposure determinants considered in the models. In this case, it would be desirable to evaluate the available information to provide a comprehensive picture of the performance of the models, and to help guide the choice of the most suitable (i.e., most reliable) model according to the process, the chemicals, and the determinants of the considered scenario. The evaluation of scenarios, chemicals, and determinants of exposure for which no evidence is available to date should be prioritized.

The results about the ECETOC TRA showed that different authors described the model as being insufficiently conservative to be a Tier1 model in several exposure scenarios (e.g., for volatile chemicals, high-dustiness chemicals, etc.), and as potentially generating false-safe scenarios. Despite most of the authors agreeing on this evaluation, few authors indicated that the model can be used as Tier1 model, but estimate results should be interpreted carefully, since overestimation or underestimation could be observed as a function of the considered scenario. Other Tier1 models (i.e., MEASE and EMKG-Expo-Tool) seem to be sufficiently conservative, but no agreement exists regarding their reliability when assessing exposure to highly volatile chemicals. Only a limited number of studies specifically evaluated these two models, so further performance analysis should be conducted.

Among the Tier2 models, STOFFENMANAGER^®^ showed a tendency to overestimate low exposure and underestimate high exposure, which, however, does not affect the model’s conservativism. Despite this, STOFFENMANAGER^®^ is the most balanced and robust model (with respect to both Tier1 and Tier2 models). This feature makes it the most suitable model for use when uncertainty persists when characterizing exposure scenarios. Despite ART showing a certain tendency to overestimate low exposures, some other studies reported underestimated exposures for some scenarios. The model was generally found to be the most accurate and precise, with a medium level of conservatism.

Other than models’ reliability, the between-users reliability was also evaluated in several studies. The results showed that inconsistency between assessors could generate widely different estimates, eventually leading to false-safe or false-unsafe scenarios. Implementations of support and quality control systems are needed to reduce potential bias among the tools’ users. TREXMO was suggested to be an effective tool for overcoming between-users and between-models biases, but further evaluations and a complete validation are still required comparing TREXMO with the original tool interfaces as golden standard. For these reasons, continuous development, adjustment, and recalibration of modeling tools are essential. The main objectives should be related to the improvement of the accuracy and precision of the models, expanding these models’ domains, and performing comprehensive validation studies.

## Figures and Tables

**Table 1:** Query used for the search in three different databases: Scopus, PubMed, and ISI Web of Knowledge (last search: 16 January 2019; weekly updates were performed until the date of submission of manuscript).

Search Query	Database
TITLE-ABS-KEY (reach) AND TITLE-ABS-KEY (“occupational exposure” OR “occupational exposure assessment” OR “occupational exposure model*” OR “exposure assessment” OR “exposure model*” OR “exposure model assessment” OR “exposure measurement” OR “exposure scenario” OR “risk assessment” OR “risk management”) AND TITLE-ABS-KEY (“ECETOC TRA” OR “ART” OR “TREXMO” OR “Stoffenmanager*” OR “Advanced REACH Tool (ART)” OR “MEASE” OR “EMKG-Expo-Tool”)	Scopus
Search ((REACH) AND ((((((((((“occupational exposure”) OR “occupational exposure assessment”) OR “occupational exposure model*”) OR “exposure assessment”) OR “exposure model*”) OR “exposure model assessment”) OR “exposure measurement”) OR “exposure scenario”) OR “risk assessment”) OR “risk management”)) AND (((((((“ECETOC TRA”) OR “ART”) OR “TREXMO”) OR “Stoffenmanager*”) OR “Advanced REACH Tool (ART)”) OR “MEASE”) OR “EMKG-Expo-Tool”)	PubMed
TS=(REACH) AND TS=(“occupational exposure” OR “occupational exposure assessment” OR “occupational exposure model*” OR “exposure assessment” OR “exposure model*” OR “exposure model assessment” OR “exposure measurement” OR “exposure scenario” OR “risk assessment” OR “risk management”) AND TS=(“ECETOC TRA” OR “ART” OR “TREXMO” OR “Stoffenmanager*” OR “Advanced REACH Tool (ART)” OR “MEASE” OR “EMKG-Expo-Tool”)	ISI Web of Knowledge

**Table 2:** Summary of statistical methods used by different authors.

Statistical Method	References
Lack of agreement between the modeling tools and the measured exposures	[21,22,25,26,28,29]
Precision	[21,22,26]
Bias, absolute/relative differences	[21,22,26,28,29]
Regression analysis and correlation between model estimates and measurements	[2,21,22,28]
Multiple linear regression analysis	[30]
Mixed-effects regression models	[23]
Logistic regression model	[32]
Ratio of exposure estimate to the measurement value	[12,33]
Calculation of percentage of measurements exceeding the exposure estimate	[12,21,33]
Comparison of the 75th and 90th percentiles of the measurement and the estimates	[12,33]
Comparison of the RCRs (risk characterization ratio) of registered ES (exposure scenario) with the observed RCRs	[14]
Evaluation of the conservatism of the tool	[8]
Uncertainty factor	[2]
Residual	[27]
Cohen κ statistics	[32]
Intraclass correlation coefficients	[32]
Evaluation of the variability of the multiple users’ outcomes	[8]
Evaluation of the choices of input parameters from the multiple users	[8]
Evaluation of which input parameters had the greatest impacts on the outcomes	[8]
Recalculation of general ventilation multipliers	[34]

**Table 3:** Summary of Process Categories (PROCs) evaluated (✓) by means of different models (STOFFENMANAGER^®^, ECETOC TRA-European Centre for Ecotoxicology and Toxicology of Chemicals Targeted Risk Assessment, and ART-Advanced Registration, Evaluation, Authorization and Restriction of Chemicals (REACH) Tool) or not evaluated (✕). Complete definition of PROCs could be found in [1,35] and in Supplementary Material (Table S2).

PROC			Model		
Code	Reference	Number of Evaluations	STOFFENMANAGER^®^	ECETOC TRA	ART
PROC1	[36]	1	✕	✓	✕
PROC2	[36]	2	✕	✓	✕
	[33]		✕	✓	✕
PROC3	[33]	1	✕	✓	✕
PROC4	[33]	4	✕	✓	✕
	[7]		✓	✓	✕
	[26]		✓	✕	✓
	[14]		✓	✓	✓
PROC5	[33]	2	✕	✓	✕
	[7]		✓	✓	✕
PROC6	—	0	—	—	—
PROC7	[37]	3	✕	✓	✓
	[7]		✓	✓	✕
	[33]		✕	✓	✕
PROC8a	[33]	2	✕	✓	✕
	[7]		✓	✓	✕
PROC8b	[33]	2	✕	✓	✕
	[7]		✓	✓	✕
PROC9	[33]	2	✕	✓	✕
	[7]		✓	✓	✕
PROC10	[36]	3	✕	✓	✕
	[7]		✓	✓	✕
	[33]		✕	✓	✕
PROC11	[7]	1	✓	—	—
PROC12	—	0	—	—	—
PROC13	[33]	2	✕	✓	✕
	[7]		✓	✓	✕
PROC14	[33]	2	✕	✓	✕
	[7]		✓	✓	✕
PROC15	[33]	1	✕	✓	✕
PROC16	—	0	—	—	—
PROC17	—	0	—	—	—
PROC18	—	0	—	—	—
PROC19	[21]	2	✓	✕	✕
	[7]		✓	✓	✕
PROC20	—	0	—	—	—
PROC21	—	0	—	—	—
PROC22	[8]	3	✓	✕	✕
	[26]		✓	✕	✓
	[14]		✓	✓	✓
PROC23	—	0	—	—	—
PROC24	—	0	—	—	—
PROC25	—	0	—	—	—
PROC26	—	0	—	—	—
PROC27a	—	0	—	—	—
PROC27b	—	0	—	—	—
PROC28	—	0	—	—	—
PROC0	[8]	29	✓	✕	✕
	[26]		✓	✕	✓
	[14]		✓	✓	✓
	[8]		✓	✕	✕
	[26]		✓	✕	✓
	[14]		✓	✓	✓
	[38]		✕	✓	✕
	[39]		✕	✕	✓
	[39]		✕	✕	✓
	[14]		✓	✓	✓
	[26]		✓	✕	✓
	[29]		✕	✕	✓
	[29]		✕	✕	✓
	[8]		✓	✕	✕
	[26]		✓	✕	✓
	[14]		✓	✓	✓
	[39]		✕	✕	✓
	[39]		✕	✕	✓
	[13]		✓	✓	✕
	[26]		✓	✕	✓
	[14]		✓	✓	✓
	[40]		✓	✕	✕
	[21]		✓	✕	✕
	[24]		✕	✕	✓
	[41]		✕	✕	✓
	[27]		✓	✓	✓
	[42]		✕	✓	✕
	[43]		✕	✓	✕
	[40]		✓	✕	✕

**Table 4:** Summary of chemicals considered in different studies.

References	Substances/Chemical Types
[7,22,26,32,44,45,46]	Powder and dust
[7,22,30,44,45]	Solids
[29,47]	Nanopowders
[7,26,32]	Liquids
[30,46]	Vapor and mist
[7,17,30,43,53]	Volatile substances
[3,14,24,33,36,37,39,42]	Organic chemicals (benzene, ethylbenzene, toluene, ethyl acetate, acetone)
[12]	Petroleum substances
[2,27,48]	Solvents
[2,13,38,39,42,49,50,51,52]	Other substances

**Table 5:** Summary of the single determinants considered in different studies.

Reference	Determinants
[41]	Activity emission potential
[41]	Substance emission potential
[41]	Localized controls
[34]	General ventilation multipliers
[51]	Ventilation rate
[51]	Room size
[51]	Amount of aerosol sprayed

**Table 6:** Summary of articles concerning the ECETOC TRA model founds in the present review.

Reference	Model Version	Scenario: Work	Scenario: Substances/Chemicals	Comments
[43]	2	Handling operations (weighing mixing, packaging, reconditioning-transferring)	Volatile substances	Few estimates were above the measured values
[12]	2	Petroleum substances		Not conservative enough for volatile liquids
[33]	3.1	Manufacturing and painting	Ethylbenzene	The model can be adequately used as a Tier1 model
[14]	3.1	Chemicals (generic)		Not very conservative
[3]	3	Toluene		The most important single factor is the PROC
[30]	3	Vapors (volatile liquids, VP > 10 Pa); Dusts; Solids (abrasive dusts).		Acceptable performances
[11]	3.1	Application of polymethylmethacrylate in floor coatings	Methyl methacrylate	Measures tends to be higher than estimates
[2]	3.1	Organic solvents, pesticides		Conservative (but not accurate) estimates
[9]	2 and 3	(To assess between-users reliability)		Not enough conservative for volatile liquids
[7]	2 and 3	Volatile substances, powders, metals, non-volatile liquids		Different performances for different chemicals
[13]	2	Professional painting indoors	1-methoxypropan-2-ol (PGME)	High variability
[38]	3	Operating room	Sevoflurane	Overestimated concentrations
[27]	3.1	Solvent cleaning tasks	Organic solvents	Low level of conservatism
[54]	n.a.	Industrial settings (wood, printing, foundry, spray painting, flour milling, chemical industry and plastic molding industry)		Not conservative enough to be a Tier1 model
[17]	2 and 3	Liquids with vapor pressure (VP) > 10 Pa		Cannot be considered conservative enough
[42]	n.a.	Plant manufacturing polyurethane foam	Mixture of isomers of TDI (mixture of toluene-2,4- or 2,6-diisocyanate) and MDI (methylene bisphenyl isocyanate)	Adequate as a Tier1 model
[37]	n.a.	Toluene		Overestimation of concentrations

**Table 7:** Summary of articles concerning the MEASE model founds in the present review.

Reference	Model Version	Scenario: Work	Scenario: Substances/Chemicals	Comments
[31]	1.02.01	24 different exposure situations (Assess between-users reliability)		—
[9]	1.02.01	(Assess between-users reliability)		—
[7]	1.02.01	Volatile substances, powders, metals, non-volatile liquids		Estimates were found higher/similar to exposure data

**Table 8:** Summary of articles concerning the EMKG model found in our review.

Reference	Model Version	Scenario: Work	Scenario: Substances/Chemicals	Comments
[31]	n.a.	25 different exposure situations		The percentage of the agent in a mixture is not considered (and this could introduce a bias)
[17]	n.a.	Exposure to liquids with vapor pressure >10 Pa		Highly conservative (except for liquids with high vapor pressure)
[11]	n.a.	Application of polymethylmethacrylate in floor coatings	methyl methacrylate	Estimates in accordance with measured exposure
[9]	n.a.	(Assess between-users reliability)		—
[7]	n.a.	Volatile substances, powders, metals, non-volatile liquids		Highly conservative for volatile liquids

**Table 9:** Summary of articles about the STOFFENMANAGER^®^ model found in the present review.

Reference	Model Version	Scenario: Work	Scenario: Substances/Chemicals	Comments
[34]	7.1	Revision of the calculations that produce the multipliers used in ART and STOFFENMANAGER^®^		General ventilation multipliers may require to be revised
[21]	n.a.	Activities belonging to two task groups: (1) handling of powders and granules and (2) machining		—
[31]	n.a	21 different exposure situations (Between-user reliability)		—
[8]	5.1	Four different types of industry: wood, printing, metal foundry, and spray painting		—
[26]	5.1	Industrial settings (wood, printing, foundry, spray painting, flour milling, chemical industry and plastic molding industry)	Handling liquids and handling powders	The model tends to overestimate situations characterized by low exposure and underestimate those with high measured exposure
[54]	5.1	Industrial settings (wood, printing, foundry, spray painting, flour milling, chemical industry, and plastic molding industry)		Modeled exposure was close to the measured exposure
[14]	6.1	Chemicals (generic)		Several false safe scenarios were detected
[27]	4.5	Liquids with VP > 10 Pa		Good accuracy, high correlation (with measured data), medium conservatism; tendency to overestimate low exposure and underestimate higher exposure
[3]	4.5	Toluene		Balanced tool
[30]	n.a.	Vapors (volatile liquids, *p* > 10 Pa), dusts, solids (abrasive dusts) and mists.		—
[22]	n.a.	Handling of: powders and granules; solids resulting in comminuting; low-volatile liquids; volatile liquids		The model can be used as a Tier1 model
[2]	6	Organic solvents and pesticide		Robust model
[9]	4.5	(Assess between-users reliability)		—
[7]	4.5	Volatile substances, powders, metals, non-volatile liquids		More conservative at higher exposure levels
[13]	4.0	Professional painting indoors, which included homogenizing and filling paint into spray gun, actual spraying and cleaning the spray gun	1-methoxypropan-2-ol (PGME)	Large variability
[25] *	6.5 and 4.0	Evaluated relative differences between STOFFENMANAGER^®^ and TREXMO (small random test)		—
[38]	5.5	Operating room (application for anesthesia purposes)	Sevoflurane	Accurate estimates
[27]	7.0	Solvent cleaning tasks	10 organic solvents: 1-bromopropane acetone acetonitrile allyl alcohol cyclohexanone glutaraldehyde 1,1-dichloro-1-fluoroethane perchloroethylene toluene trichloroethylene	Balanced model: good accuracy, high correlation, medium conservatism
[53]	4.5	19 different workplaces		Model is reasonably accurate and robust for what concern estimates of liquids with VP > 10 Pa
[52]	7.1	Packing of an inorganic complex fertilizer in an industrial plant	Inorganic complex fertilizer	The tool tends to overestimate the exposure level
[44]	5.1	Dust, abrasive dust (solid), and liquid		—
[56]	5.0	Different exposure scenarios in medium-sized enterprises		Can be used in intervention studies
[40]	7.1	Packing of ceramic materials	Ceramic materials (clays, feldspars, kaolin and quartz)	The model tends to overestimate concentrations

* This paper was not retrieved within results of the research in scientific databases but included here for discussion.

**Table 10:** Summary of articles found in the present review concerning the ART model.

Reference	Model Version	Scenario: Work	Scenario: Substances/Chemicals	Comments
[29]	n.a.	Dumping and dumping + mixing of nanopowders	Handling of ~100% nanopowders (TiO_2_, Al_2_O_3_, and SiO_2_).	The model overestimates exposure at low concentrations
[23]	n.a.	(Development of the mechanistic model)		—
[34]	1.5	(Revision of the calculations that produce the multipliers used in ART and STOFFENMANAGER^®^)		General ventilation multipliers may require revision
[26]	1.5	Industrial settings (wood, printing, foundry, spray painting, flour milling, chemical, and plastic molding industries)	Handling liquids and handling powders	Tends to underestimate the exposure and have a lower agreement in wood industry activities; handling liquids activities showed higher agreement than situations involving handling of powders
[14]	n.a.	Industrial settings (wood, printing, foundry, spray painting, flour milling, chemical, and plastic molding industries)		ART may underestimate the exposure in general. Low number of false safe scenarios
[14]	1.5	Chemicals (generic)		ART should be used with caution for chemicals with high VP and low DNELs (Derived No Effect Level)
[24]	1.5	Use of a metal parts washer	Benzene	Bayesian module that can be applied to the mechanistic model
[53]	1.5	19 different workplaces		Accurate for liquids with VP > 10 Pa; underestimate exposure for different tasks
[41]	n.a.	Pharmaceutical company		In most scenarios investigated, GM exposure estimates were lower than measured exposure level
[39]	1.5	Spraying of antifouling paints and shoe repair	Copper pyrithione + ethyl acetate	—
[3]	1.5	Toluene		Most important and influence factors are local controls and the source emission
[49]	n.a.	Welding fumes		Modifying factors to be amended to include welding fumes in model’s domain
[45]	n.a.	Powders, vapors, solids		The 90th percentile showed sufficient conservatism. The model tends to overestimate lower exposure and underestimate high exposure levels
[30]	1.5	Vapors (volatile liquids, pressure > 10 Pa), dusts, solids (abrasive dusts) and mists.		—
[46]	n.a.	Stratified analyses were conducted for different forms of exposure (abrasive dust, dust, vapors, and mists).		Calibration of the model
[58]	1.5	(Describe the structures and functionalities of the ART exposure database)		—
[32]	1.0	Liquid and dust scenario		—
[2]	1.5	Organic solvents and pesticide		Accurate estimations; tends to underestimate the exposure for pesticides
[57]^*^	1.0	(General outline of ART)		Mechanistic model, exposure prediction, applicability domain of the model
[59]	1.0	Handling of: powders, granules, and pellets resulting in dust exposure; solid objects resulting in dust exposure; (volatile) liquids resulting in exposure to vapor; (low volatility) liquids resulting in exposure to mists		—
[27]	1.5	Solvent cleaning tasks	10 organic solvents: 1-bromopropane acetone acetonitrile allyl alcohol cyclohexanone glutaraldehyde 1,1-dichloro-1-fluoroethane perchloroethylene toluene trichloroethylene	Most accurate and precise; medium conservatism
[37]	n.a.	Toluene		High agreement with experimental results
[40]	n.a.	Packing of ceramic materials	Ceramic materials (clays, feldspars, kaolin and quartz)	The model overestimated exposure concentration

* This paper was not retrieved within results of the research in scientific databases but included here for discussion.

**Table 11:** Summary of articles found about the TREXMO tool for the present review.

Reference	Model Version	Objective of the Study
[5]	1	Development, validation, and performance of the Translation Exposure Models (TREXMO)
[25] *	1	Evaluated relative differences between STOFFENMANAGER^®^ 6.5 and TREXMO (small random test)
[44]	1	Comparison between STOFFENMANAGER^®^ 6 and TREXMO
[30]	1	Evaluation of exposure models (ART, STOFFENMANAGER^®^ 4.0, and ECETOC TRA) via correlation and consistency analysis performed with TREXMO
[60]	1	Inter-assessor agreement for TREXMO (MEASE v. 1.02.01; EMKG-Expo-Tool; ECETOC TRA v.3; STOFFENMANAGER^®^ 4.0; ART v.1.5)

* This paper was not retrieved within results of the research in scientific databases but included here for discussion.

## Supplementary Materials

The following are available online at https://www.mdpi.com/1660-4601/16/15/2764/s1, Table S1: Complete list of papers found suitable and used in this review; Table S2: Definition of PROCs.
