# Supplementary material for: How to Obtain a Reliable Estimate of Occupational Exposure? Review and Discussion of Models’ Reliability
Source: Int J Environ Res Public Health. 2019 Aug 2;16(15):2764. doi: 10.3390/ijerph16152764 (PMC6695664; doi:10.3390/ijerph16152764)
Supplement: Supplementary file 1 [file ijerph-16-02764-s001.pdf]

International Journal of
Environmental Research and Public Health

MDPI

Review

How to Obtain a Reliable Estimate of Occupational Exposure? Review and Discussion of Models' Reliability

Andrea Spinazzè *,†, Francesca Borghi *,†, Davide Campagnolo, Sabrina Rovelli, Marta Keller, Giacomo Fanti, Andrea Cattaneo and Domenico Maria Cavallo

Dipartimento di Scienza e Alta Tecnologia, Università degli Studi dell'Insubria, Via Valleggio 11, 22100 Como, Italy
* Correspondence: andrea.spinazze@uninsubria.it (A.S.); f.borghi2@uninsubria.it (F.B.); Tel.: +39-031-238-6629 (A.S. & F.B.)
† These authors contributed equally to this work.

Received: 3 July 2019; Accepted: 30 July 2019; Published: date

Table S1: Complete List of Papers Found Suitable and Used in This Review

First Author	Title	Source Title	Volume	Issue	Year	DOI
Angelini	Respiratory Health – Exposure Measurements and Modeling in the Fragrance and Flavour Industry	PLOS ONE			2016	10.1371/journal.pone.0148769
Bekker	The validity and applicability of using a generic exposure assessment model for occupational exposure to nano-objects and their aggregates and agglomerates	Annals of Occupational Hygiene	60	9	2016	10.1093/annhyg/mew048
Fransman	Advanced reach tool (ART): Development of the mechanistic model	Annals of Occupational Hygiene	55	9	2011	10.1093/annhyg/mer083
Fransman	How Accurate and Reliable Are Exposure Models?	Annals of Work Exposure and Health	61	8	2017	10.1093/annweh/wxx068
Hesse	Review of Tier 1 workplace exposure estimates for petroleum substances in REACH dossiers	CONCAWE Reports		13	2018	n.a.
Hofstetter	Evaluation of Recommended REACH Exposure Modeling Tools and Near-Field, Far-Field Model in Assessing Occupational Exposure to Toluene from Spray Paint	Annals Occupational Hygiene	57	2	2013	10.1093/annhyg/mes062
Ishii	Evaluation of the ECETOC TRA model for workplace inhalation exposure to ethylbenzene in Japan	Journal of Chemical Health&Safety	24	1	2017	10.1016/j.jchas.2016.03.003
Jankowska	Application of predictive models for estimation of health care workers	International Journal of Occupational Safety and Ergonomics	21	4	2015	10.1080/10803548.2015.1086183

	exposure to sevoflurane					
Koivisto	The general ventilation multipliers calculated by using a standard Near-Field/Far-Field model	Journal of Occupational and Environmental Hygiene			2018	10.1080/15459624.2018.1440084
Koppisch	Use of the MEGA Exposure Database for the Validation of the Stoffenmanager Model	Annals of Occupational Hygiene	56	4	2012	10.1093/annhyg/mer097
Kupczewska-Dobecka	Evaluation of the TRA ECETOC model for inhalation workplace exposure to different organic solvents for selected process categories	International Journal of Occupational Medicine and Environmental Health	24	2	2011	10.2478/s13382-011-0021-3
Kupczewska-Dobecka	Assessment of exposure to TDI and MDI during polyurethane foam production in Poland using integrated theoretical and experimental data	Environmental toxicology and pharmacology	34		2012	10.1016/j.etap.2012.06.006
Lamb	Between-User Reliability of Tier 1 Exposure Assessment Tools Used Under REACH	Annals of Work Exposure and Health	61	8	2017	10.1093/annweh/wxx074
Landberg	A Study of the Validity of Two Exposure Assessment Tools: Stoffenmanager and the Advanced REACH Tool	Annals of Work Exposure and Health	61	5	2017	10.1093/annweh/wxx008
Landberg	Comparison and Evaluation of Multiple Users' Usage of the Exposure and Risk Tool: Stoffenmanager 5.1	Annals of Occupational Hygiene	59	7	2014	10.1093/annhyg/mev027
Landberg	Evaluating the Risk Assessment Approach of the REACH Legislation: A Case Study	Annals of Work Exposure and Health	63	1	2019	10.1093/annweh/wxy090

Landberg	Evaluation of risk assessment approaches of occupational chemical exposures based on models in comparison with measurements	Safety Science	109		2018	10.1016/j.ssci.2018.06.006
LeBlanc	Comparison of the near field/far field model and the advanced reach tool (ART) model V1.5: exposure estimates to benzene during parts washing with mineral spirits	International Journal of Hygiene and Environmental Health	221	2	2018	10.1016/j.ijheh.2017.10.016
Lee	Evaluation of Exposure Assessment Tools under REACH: Part II-Higher Tier Tools	Annals of Work Exposure and Health	9		2018	10.1093/annweh/wxy098
Lee	Evaluation of Exposure Assessment Tools under REACH: Part I-Tier 1 Tools	Annals of Work Exposure and Health	9		2018	10.1093/annweh/wxy091.
Lee	Comparison of Quantitative Exposure Models for Occupational Exposure to Organic Solvents in Korea	Annals of Work Exposure and Health			2018	10.1093/annweh/wxy087
Mc Donnell	Validation of the inhalable dust algorithm of the Advanced REACH Tool using a dataset from the pharmaceutical industry	Journal of Environmental Monitoring	13	6	2011	10.1039/c1em10189g
McNally	Advanced REACH tool: A Bayesian model for occupational exposure assessment	Annals of Occupational Hygiene	58	5	2014	10.1093/annhyg/meu017
Mostert	REACH Worker Exposure Model for Co-formulants Used in Plant Protection Products	Annals of Work Exposure and Health	63	1	2019	10.1093/annweh/wxy088
Park	Comparison of modeled estimates of inhalation exposure to aerosols during use	International Journal of Hygiene and Environmental Health	221	6	2018	10.1016/j.ijheh.2018.05.005

	of consumer spray products					
Ribalta	Testing the performance of one and two box models as tools for risk assessment of particle exposure during packing of inorganic fertilizer	Science of the Total Environment	650		2019	10.1016/j.scitotenv.2018.09.379
Riedmann	Sensitivity analysis, dominant factors, and robustness of the ECETOC TRA v3, Stoffenmanager 4.5, and ART 1.5 occupational exposure models	Risk Analysis	35	2	2015	10.1111/risa.12286
Sailabaht	Extension of the advanced REACH tool (ART) to include welding fume exposure	International Journal of Environmental Research and Public Health	15	10	2018	10.3390/ijerph15102199
Savic	ART, stoffenmanager, and TRA: A systematic comparison of exposure estimates using the TREXMO translation system	Annals of Work Exposures and Health	62	1	2018	10.1093/annweh/wxx079
Savic	Comparing the Advanced REACH Tool's (ART) estimates with Switzerland's occupational exposure data	Annals of Work Exposures and Health	61	8	2018	10.1093/annweh/wxx069
Savic	TREXMO: A Translation Tool to Support the Use of Regulatory Occupational Exposure Models	Annals of Occupational Hygiene	60	8	2016	10.1093/annhyg/mew042
Savic	Inter-assessor Agreement for TREXMO and Its Models Outside the Translation Framework	Annals of Work Exposures and Health			2019	10.1093/annweh/wxz040
Schinkel	Advanced REACH Tool (ART): Calibration of the mechanistic model	Journal of Environmental Monitoring	13	5	2011	10.1039/c1em00007a

Schinkel	Cross-validation and refinement of the Stoffenmanager as a first tier exposure assessment tool for REACH	Occupational and Environmental Medicine	67	2	2010	10.1136/oem.2008.045500
Schinkel	Reliability of the Advanced REACH Tool (ART)	Annals of Occupational Hygiene	58	4	2014	10.1093/annhyg/met081
Schinkel	The advanced REACH tool (ART): Incorporation of an exposure measurement database	Annals of Occupational Hygiene	57	6	2013	10.1093/annhyg/mes103
Spee	Comparing REACH Chemical Safety Assessment information with practice a case-study of polymethylmethacrylate (PMMA) in floor coating in The Netherlands	International Journal of Hygiene and Environmental Health	220	7	2017	10.1016/j.ijheh.2017.05.012
Spinazzè	Accuracy evaluation of three modelling tools for occupational exposure assessment	Annals of Work Exposures and Health	61	3	2017	10.1093/annweh/wxx004
Terwoert	An Intervention Study on the Implementation of Control Banding in Controlling Exposure to Hazardous Chemicals in Small and Medium-sized Enterprises	Safety and Health at Work	7		2016	10.1016/j.shaw.2015.12.002
Tielemans	Advanced REACH Tool (ART): Overview of version 1.0 and research needs	Annals of Occupational Hygiene	55	9	2011	10.1093/annhyg/mer094
Tielemans	Development of an advanced exposure assessment tool: Advanced REACH Tool (ART)	Journal of Physics Conference Series	151		2009	

Tischer	Evaluation of Tier One Exposure Assessment Models (ETEAM): Project Overview and Methods	Annals of Work Exposures and Health	61	8	2017	10.1093/annweh/wxx066
Van Tongeren	Advanced REACH Tool: Development and application of the substance emission potential modifying factor	Annals of Occupational Hygiene	55	9	2011	10.1093/annhyg/mer093
Van Tongeren	Validation of lower tier exposure tools used for REACH: Comparison of tools estimates with available exposure measurements	Annals of Work Exposures and Health	61	8	2017	10.1093/annweh/wxx056
Vink	Use of read-across and tiered exposure assessment in risk assessment under REACH - A case study on a phase-in substance	Regulatory Toxicology and Pharmacology	58	1	2010	10.1016/j.yrtph.2010.04.004

Table S2: Definition of PROCs

**PROC Process Category**	
**Code**	**Name**
PROC1	Chemical production or refinery in closed process without likelihood of exposure or processes with equivalent containment conditions
PROC2	Chemical production or refinery in closed continuous process with occasional controlled exposure or processes with equivalent containment conditions
PROC3	Manufacture or formulation in the chemical industry in closed batch processes with occasional controlled exposure or processes with equivalent containment condition
PROC4	Chemical production where opportunity for exposure arises
PROC5	Mixing or blending in batch processes
PROC6	Calendering operations
PROC7	Industrial spraying

PROC8a	Transfer of substance or mixture (charging and discharging) at non-dedicated facilities
PROC8b	Transfer of substance or mixture (charging and discharging) at dedicated facilities
PROC9	Transfer of substance or mixture into small containers (dedicated filling line, including weighing)
PROC10	Roller application or brushing
PROC11	Non industrial spraying
PROC12	Use of blowing agents in manufacture of foam
PROC13	Treatment of articles by dipping and pouring
PROC14	Tabletting, compression, extrusion, pelletisation, granulation
PROC15	Use as laboratory reagent
PROC16	Use of fuels
PROC17	Lubrication at high energy conditions in metal working operations
PROC18	General greasing /lubrication at high kinetic energy conditions
PROC19	Manual activities involving hand contact
PROC20	Use of functional fluids in small devices
PROC21	Low energy manipulation and handling of substances bound in/on materials or articles
PROC22	Manufacturing and processing of minerals and/or metals at substantially elevated temperature
PROC23	Open processing and transfer operations at substantially elevated temperature
PROC24	High (mechanical) energy work-up of substances bound in /on materials and/or articles
PROC25	Other hot work operations with metals
PROC26	Handling of solid inorganic substances at ambient temperature
PROC27a	Production of metal powders (hot processes)
PROC27b	Production of metal powders (wet processes)
PROC28	Manual maintenance (cleaning and repair) of machinery
PROC0	Other
